# Clinical efficacy and safety of ultrasound-assisted thrombolysis vs. standard catheter-directed thrombolysis in patients with acute pulmonary embolism: A study level meta-analysis of clinical trials

**DOI:** 10.3389/fcvm.2022.967786

**Published:** 2022-10-05

**Authors:** Bing Sun, Jing Xiao Yang, Zi Kuan Wang, Hai Jia Zhou, Yi Chu, Yan Li, Rui Rui Chen

**Affiliations:** Department of Cardiology, Tangdu Hospital, Air Force Medical University, Xi'an, China

**Keywords:** acute pulmonary embolism, catheter-directed thrombolysis, tissue plasminogen activator, ultrasound-assisted thrombolysis, standard catheter-directed thrombolysis

## Abstract

**Aim:**

To compare the clinical efficacy of ultrasound-assisted thrombolysis (USAT) vs. standard catheter-directed thrombolysis (SCDT) in patients with acute pulmonary embolism (aPE).

**Methods:**

This study analyzed the clinical outcomes of patients with non-low-risk aPE who received USAT or SCDT. The primary outcomes were all-cause death, total bleeding, and major bleeding. Secondary outcomes included pulmonary thrombotic load score (Miller), improvement in right ventricular-to-left ventricular ratio (RV/LV), dose and duration of the thrombolytic drug tissue plasminogen activator (tPA), length of stay (LOS) in the ICU, and total LOS in the hospital.

**Results:**

A total of seven articles and 451 patients were included in this study. 241 patients were in the USAT group and 210 patients were in the SCDT group. There were no significant differences in all-cause mortality, total bleeding, and major bleeding between the two groups. Miller scores for pulmonary thrombus also showed no difference between the two groups, but pulmonary artery systolic pressure (PASP) was lower in the SCDT group after-treatment. The reduction of RV/LV from baseline was more pronounced in the SCDT group than in the USAT group (OR: −0.14, 95%CI: −0.20 to 0.07, *P* < 0.0001, *I*^2^ = 0%). Total dose of tPA and duration of infusion in the USAT group were lower than those in the SCDT group, but there was no significant statistical difference. LOS in the ICU was similar between the two groups, while LOS in the hospital was lower in the SCDT group.

**Conclusion:**

This study did not detect any differences in all-cause mortality, total bleeding, and major bleeding between non-low-risk aPE patients treated with USAT or SCDT. Improvement in right ventricular function was better in the SCDT group, and hospital LOS was lower in the SCDT group.

## Introduction

Pulmonary Embolism (PE) has become the third most common cause of death in cardiovascular diseases, after only myocardial infarction and stroke (1). The global incidence of PE is 39-115/100,000, and these rates have steadily increased in recent years (2–4). Anticoagulation and systemic thrombolysis are the recommended antithrombotic strategies by guidelines; however, they each have some limitations. Anticoagulation alone (AC) cannot rapidly reduce pulmonary thrombotic load and improve right ventricular function, while systemic thrombolysis (ST) significantly increases the risk of bleeding (5–9). Therefore, a more effective and safe treatment strategy is needed for PE patients. Meta-analysis showed that Catheter-directed thrombolysis (CDT) reduced the 30-day and 1-year all-cause mortality in submassive pulmonary embolism (sPE) patients compared to AC (10), and reduced the all-cause mortality during hospitalization, and incidence of intracranial hemorrhage compared to ST (11). In addition, CDT can better prevent the development of chronic thromboembolic pulmonary hypertension in aPE patients. These studies suggest CDT may be an attractive treatment option for aPE (12).

Presently, there are two types of thrombolysis catheters commonly used in CDT, those used in ultrasound-assisted thrombolysis (USAT) and those used in standard catheter-directed thrombolysis (SCDT). The USAT thrombolytic catheter was approved by the FDA in 2014 and is the only thrombolytic catheter approved by the FDA to treat PE. The catheter consists of a cooling chamber, drug delivery chamber, ultrasonic core cavity, and two spare chambers. The ultrasonic core cavity not only helps with blood clots, but can also change the fibrous protein structure, which exposing more of the drug binding sites and enhancing the efficiency of thrombolysis, while drug delivery chamber was used to delve drugs (13–16). The catheter used in SCDT is a standard thrombolytic catheter made by Medtronic or AngioDynamics with a relatively simple structure consisting of multiple lateral channels. Thrombolytic agent is released through the lateral hole to achieve *in situ* thrombolysis. Theoretically, the use of ultrasound gives USAT a higher thrombolytic efficiency than SCDT, which is manifested by a lower total duration and dosage of thrombolytic agent. However, clinical trials have been controversial on this idea (17–23). Here, we performed a meta-analysis of published clinical trials that directly compared USAT and SCDT for aPE, in order to seek the appropriate thrombolysis catheter for patients with aPE.

## Methods

### Inclusion and exclusion criteria

Inclusion criteria: (1) Identification of PE by computed tomography angiography (CTA); (2) Right ventricular dysfunction (RV/LV diameter ratio >0.9) or increased markers of myocardial injury (troponin or brain natriuretic peptide BNP) evident from CTA or echocardiography. Exclusion criteria: age <18 years, symptom duration >14 days, signs of shock and hypotension (systolic blood pressure <90 mmHg or a decrease of ≥40 mmHg from baseline for more than 15 min), high risk of bleeding (including previous intracranial hemorrhage, known structural intracranial cerebrovascular disease or tumor, stroke within the previous 3 months, suspected aortic dissection, active bleeding, recent spinal or cranial/brain surgery, recently closed head or facial trauma accompanied with a bone fracture or brain injury).

### Intervention measures and outcomes

Ultrasound-assisted thrombolysis group received treatment using a thrombolytic catheter made by EkoSonic Endovascular System (EKOS). SCDT group received treatment using standard thrombolytic catheters made by Medtronic or AngioDynamics. Primary endpoints were all-cause mortality, incidence of bleeding, and major bleeding. Secondary endpoints were pulmonary thrombotic load score (Miller), post-operative pulmonary artery systolic blood pressure, improvement in right ventricular function (RV/LV diameter ratio), dose time of the thrombolytic drug tissue plasminogen activator (tPA), LOS in the ICU, and total LOS in the hospital.

### Search strategy

The present work collected clinical trials from four databases, including PubMed, EMBASE, Cochrane Library, and Clinical Trials, that were searched by computer from January 1st, 2012 to May 1st, 2022. A combination of Medical Subject Heading and EMTREE terms were used, including “ultrasound-assisted catheter-directed thrombolysis, ultrasound-assisted thrombolysis, ultrasound-accelerated thrombolysis, standard catheter-directed thrombolysis, pulmonary embolism, lung pulmonary embolism, pulmonary thromboembolism.” Retrospective reference of the included literature was also performed to supplement the acquisition of relevant literature. The data from the included articles were extracted independently by two investigators, and any discrepancies were resolved by a third investigator. The extracted data included: (1) article information, including the publication year and names of the authors; and (2) procedural characteristics, including study type, sample size, interventional measures, follow-up time, and all relative outcomes. The quality of the included literature was determined by Cochrane evaluation tools for randomized controlled trials (RCT) and by NOS scales for non-randomized controlled studies.

### Statistical methods

RevMan 5.4 and Stata 17.0 statistical software were adopted for performing a meta-analysis. The heterogeneity of involved articles was explored by the χ^2^ test (test level a = 0.1) and evaluated by *I*^2^ statistics. The fixed-effect model was nurtured for meta-analysis if the heterogeneity test results were *I*^2^ < 50%. If the consistency test results were *I*^2^ ≥ 50%, the random effect model was applied for meta-analysis. Sensitivity analysis was conducted after removing single studies in turn. The publication bias was tested by Egger's regression.

## Results

### Literature screening

**Figure 1:**
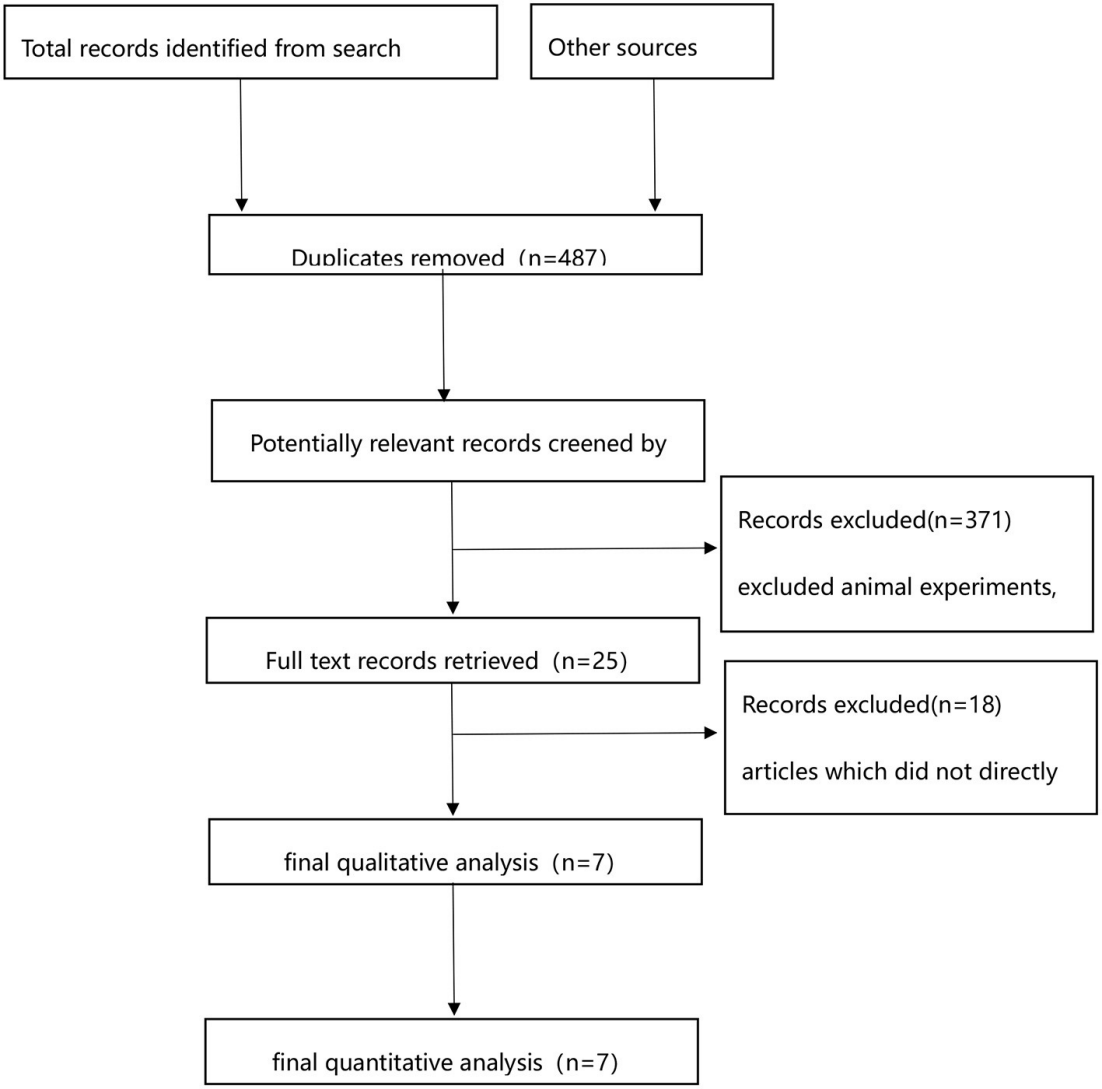
PRISMA flow diagram.

A total of 571 publications were returned after database screening, and 93 duplicates were excluded. Animal experiments, case reports, reviews, meta-analyses, and conference proceedings were also excluded. The remaining 25 articles were re-screened, and there was no direct comparison between USAT and SCDT in 18 of them. Finally, seven articles were included, as illustrated in Figure 1.

### Article information and procedural characteristics

Table 1 presents article information and procedural characteristics, including author, publication year, study type, sample size, intervention method, antithrombotic strategy, primary and secondary outcomes, and follow-up time.

**Table 1:** Baseline and procedural characteristics.

**Author**	**Study type**	**Sample size**		**Thrombolysis drugs**	**Outcomes**	**Follow-up time**
		**USAT group**	**SCDT group**			
Avgerinos et al. (23)	RCT	40	41	USAT: tPA, 19 ± 7 mg,14 ± 5 h SCDT: tPA, 18 ± 7 mg, 14 ± 6 h; no loading dose, max tPA <24 mg	Death, bleeding, major bleeding, millers score, RV/LV diameter ratio, PAMP, ICU and hospital stay	3 months, 12 months
Allen et al. (22)	Non-RCT	31	23	USAT: tPA, 20 ± 0.8 mg, 12 ± 0.4 h SCDT: tPA, 23 ± 0.75 mg, 11 ± 0.5 h A bolus dose, continuous infusion 1 mg/h *via* both catheters.	Death, bleeding, major bleeding, RV/LV diameter ratio, PAMP	4 weeks
Rao et al. (20)	Non-RCT	37	33	USAT: tPA, 25.1 ± 6.3 mg, 13.3 ± 3.2 h SCDT: tPA, 24.7 ± 12.2 mg, 13.3 ± 3.9 h; bolus dose 2–5 mg, continuous infusion 0.5–1 mg/h through each catheter	Death, bleeding, major bleeding, RV/LV diameter ratio, PASP, hospital stay	Hospitalization 18 months
Rothschild et al. (21)	Non-RCT	62	36	USAT: tPA, 34.4 ± 17.3 mg, 31.5 ± 17.3 h SCDT: tPA, 28.7 ± 14 mg, 22.9 ± 14.5 h; bolus dose 5 mg, continuous infusion 0.5–1 mg/h through each catheter	Death, bleeding, major bleeding, RV/LV diameter ratio, PASP, ICU and hospital stay	Hospitalization
Graif et al. (19)	Non-RCT	24	36	USAT: tPA, 27.1 ± 11.3 mg, 23.9 ± 8.8 h SCDT: tPA, 33.6 ± 13.9 mg, 30.4 ± 12.6 h; no loading dose, continuous infusion 0.25–1 mg/h through each catheter	Death, bleeding, major bleeding, PASP, PAMP, millers score	30 days
Liang et al. (18)	Non-RCT	36	27	USAT: tPA, 23.2 ± 13.7 mg, 15.4 ± 5.3 h SCDT: tPA, 23.7 ± 13.9 mg, 13.3 ± 3.9 h; bolus dose 2–4 mg, continuous infusion 0.5–1 mg/h through each catheter	Death, bleeding, major bleeding, ICU stay	3 months 12 months
Lin (17)	Non-RCT	11	15	USAT: tPA, 17.2 ± 2.36 mg, 17.4 ± 5.23 h SCDT: tPA, 25.43 ± 5.27 mg, 26.7 ± 8.64 h; no loading dose, continuous infusion 0.86 mg/h in USAT, 0.93 mg/h in SCDT	Death, bleeding, major bleeding, millers score	Hospitalization

### Quality assessment

The present study included seven articles, including one randomized controlled trial (RCT) and six non-randomized controlled studies. The Cochrane evaluation tool was employed for the RCT, while NOS scales were applied for the non-randomized controlled studies to evaluate the quality of the covered literature. The quality evaluation of the RCT trial literature reflected high quality. The Newcastle-Ottawa Scale (NOS) evaluation results of the non-randomized controlled study were discussed in Table 2, and three studies are six points, and another three studies are five, seven and eight points, respectively.

**Table 2:** Quality assessment.

**Author**	**Selection of case (4 scores)**				**Comparison between experimental**	**Methods of exposure**	
					**group and control group (2 scores)**	**assessment (4 scores)**	
	**Definition and diagnosis of cases**	**Representativeness of cases**	**Control selection**	**Definition of control**		**Methods of investigation and assessment (2 scores)**	**Investigation no response rates**
Allen et al. (22)	√	√	√	√	√	√	√
Rao et al. (20)	√		√	√	√		√
Rothschild et al. (21)	√		√	√		√	√
Graif et al. (19)	√		√		√		√
Liang et al. (18)	√		√	√		√	√
Lin (17)	√	√	√	√		√	√

## Outcomes

### Primary outcomes

#### All-cause death, incidence of bleeding, and major bleeding

**Figure 2:**
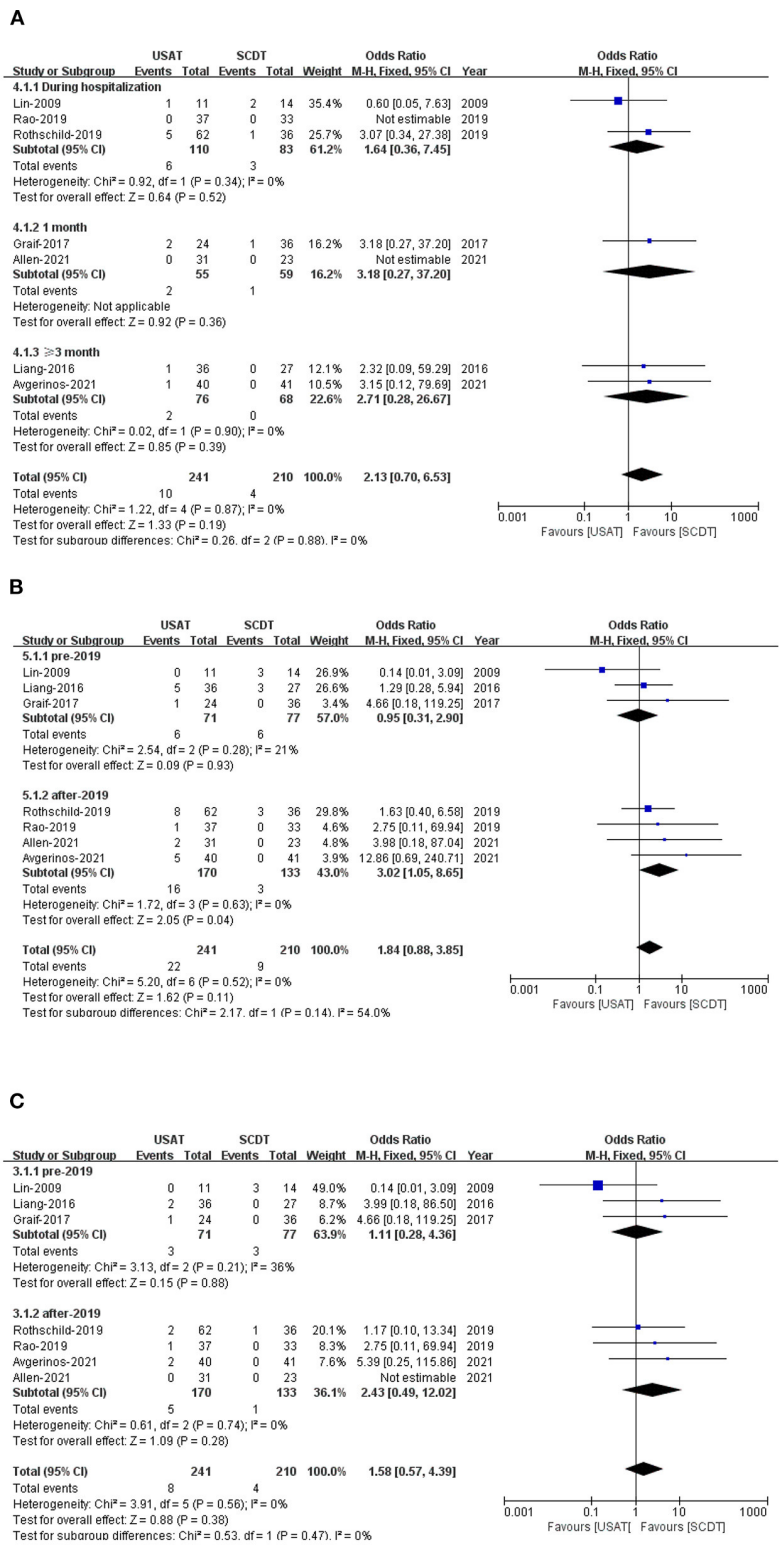
**(A)** Forest of all-cause death. **(B)** Forest of total bleeding. **(C)** Forest of major bleeding.

All-cause mortality was 4.1% in the USAT group and 1.9% in the SCDT group (OR: 2.13, 95%CI: 0.70–6.53, *P* = 0.19, *I*^2^ = 0%) (Figure 2A). Incidence of bleeding was 9.1% in the USAT group and 4.3% (OR: 1.84, 95%CI: 0.88–3.85, *P* = 0.11, *I*^2^ = 0%) in the SCDT group (Figure 2B), and major bleeding was 3.3% in the USAT group and 1.9% in the SCDT group (OR: 1.58, 95%CI: 0.57–4.39, *P* = 0.38, *I*^2^ = 0%). The differences were not statistically significant in these categories (Figure 2C). However, after-2019 USAT group has higher incidence of bleeding than SCDT group (9.4 vs. 2.3%, OR: 3.02, 95%CI: 1.05–8.65, *P* = 0.04, *I*^2^ = 0%) (Figure 2B).

### Secondary outcomes

#### RV/LV diameter ratio

**Figure 3:**
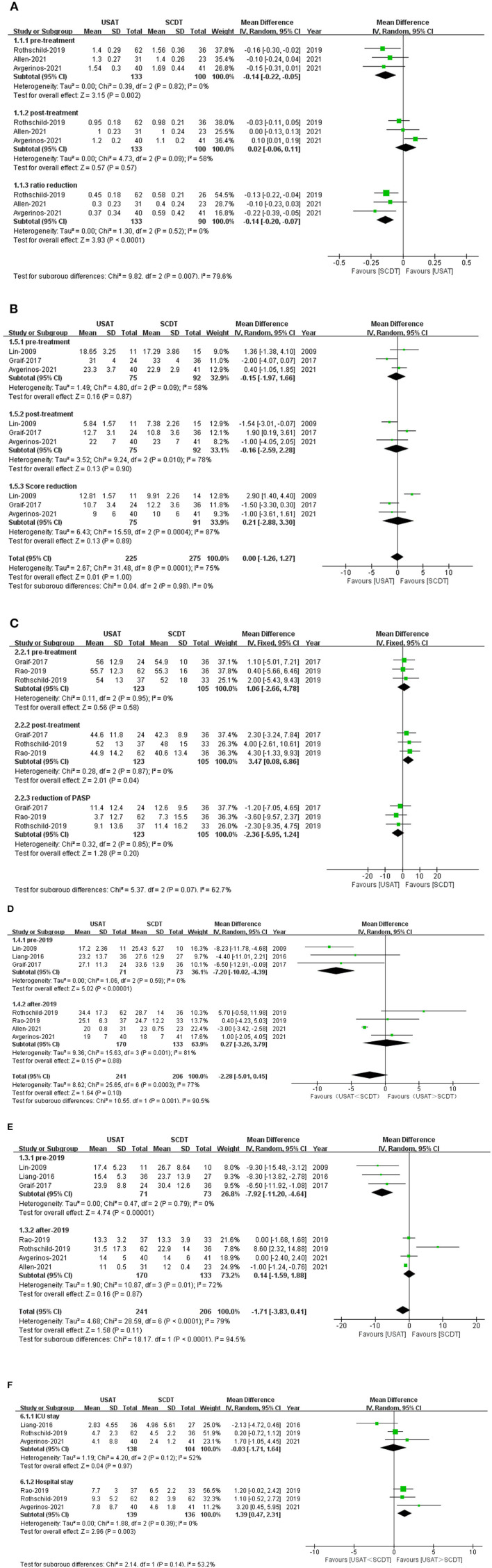
**(A)** Forest of right ventricular-to-left ventricular ratio (RV/LV). **(B)** Forest of millers score. **(C)** Forest plot of pulmonary arterial systolic pressure (PASP). **(D)** Forest plot of dose of tPA. **(E)** Forest plot of time of tPA. **(F)** Forest plot of ICU and lenth of stay (LOS) in the hospital.

Right ventricular-to-left ventricular ratio was lower in the USAT group than in the SCDT group before treatment (OR: −0.14, 95%CI: −2.2 to 0.05, *P* = 0.002, *I*^2^ = 0%), but there was no significant difference between the two groups after treatment (OR: −0.02, 95%CI: −0.06 to 0.11, *P* = 0.57, *I*^2^ = 58%). RV/LV reduction (OR: −0.14, 95%CI: −0.20 to 0.07, *P* < 0.0001, *I*^2^ = 0%) was greater in the SCDT group than in the USAT group (Figure 3A).

#### Pulmonary thrombotic load score (miller score) and pulmonary artery systolic pressure (PASP)

There were no statistical significance before, after treatment and reduction of Millers Score, before treatment USAT group vs. SCDT group (OR: −0.12, 95%CI: −1.2 to 0.97, *P* = 0.84, *I*^2^ = 58%), after treatment (OR: −0.18, 95%CI: −1.23 to 0.86, *P* = 0.73, *I*^2^ = 58%), reduction (OR: 0.21, 95%CI: −2.88 to 3.30, *P* = 0.89, *I*^2^ = 87%) (Figure 3B). There were no difference in pre-treatment (OR: 1.06, 95%CI: −2.66 to 4.78, *P* = 0.95, *I*^2^ = 0%) and reduction of PASP (OR: −2.36, 95%CI: −5.95 to 1.24, *P* = 0.20, *I*^2^ = 0%) between two groups. But PASP after-treatment in the USAT group was higher than in the SCDT group (OR: 3.47, 95%CI: 0.08–6.86, *P* = 0.04, *I*^2^ = 0%), and the difference was statistically significant (Figure 3C).

#### Dose and duration of tPA

Duration of thrombolytic tPA administration (OR: −1.71, 95%CI: −3.83 to 0.41, *P* = 0.11, *I*^2^ = 79%) and dose of thrombolytic tPA (OR: −2.28, 95%CI: −5.01 to 0.45, *P* = 0.10, *I*^2^ = 77%) showed no statistical differences between the groups. Before 2019, the duration of thrombolytic tPA administration (OR: −7.92, 95%CI: −11.20 to 4.64, *P* < 0.0001, *I*^2^ = 0%) and the dose of thrombolytic drugs (tPA) (OR: −7.20, 95%CI: −10.02 to 4.93, *P* < 0.00001, *I*^2^ = 0%) were both lower in the USAT group than SCDT group. After 2019, no significant differences were found in either the duration of thrombolytic tPA administration (OR: 0.14, 95%CI: −1.59 to 1.88, *P* = 0.87, *I*^2^ = 72%) or the dose of thrombolytic tPA (OR: 0.27, 95%CI: −3.26 to 3.79, *P* = 0.32, *I*^2^ = 85%) (Figures 3D,E).

#### ICU and LOS

There was no significant difference between two groups on Length of stay in the ICU (OR: −0.03, 95%CI: −1.71 to 1.64, *P*=0.97, *I*^2^ = 52%). Total length of stay (LOS) in the hospital was significantly higher in the USAT group than in the SCDT group (OR: 1.39, 95%CI: 0.47–2.31, *P* = 0.003, *I*^2^ = 85%) (Figure 3F).

#### Sensitivity analysis

**Figure 4:**
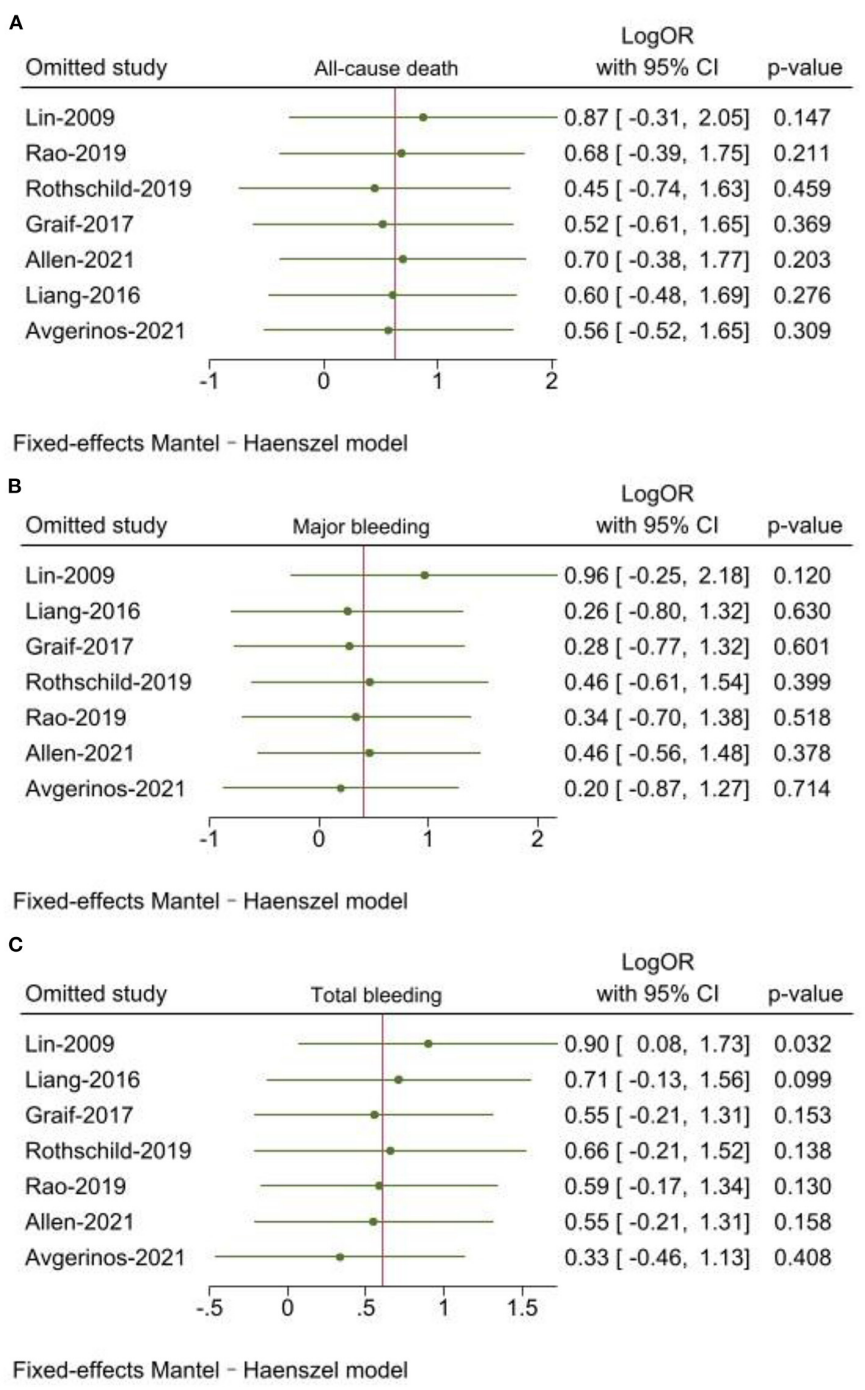
**(A–C)** Sensitivity analysis of all-cause death, major bleeding and total bleeding.

Leave-one-out sensitivity analyses was used by Stata 17.0. The results of all-cause death and major bleeding were consistent with main analysis. When Lin (17) was excluded, there was a statistically significant difference for the outcome of total bleeding (*P* = 0.032) (Figures 4A–C).

#### Publication bias

Publication bias was analyzed for all-cause death by Egger's regression (*P* = 0.6249). No publication bias was found in our study.

## Discussion

All-cause mortality, incidence of total bleeding and major bleeding were measured in all seven articles included. Both the USAT and SCDT groups had shown low mortality rates during hospitalization (5.5 vs. 3.6%), within 1 month (3.6 vs. 1.7), and over 3 months (2.6 vs. 0%), which are consistent with existing clinical trials (24, 25). USAT group seem to have a trend of higher all-cause death, although the difference was not significantly compared to SCDT. Lower trend of all-cause death in SCDT group might be associated with lower major bleeding (1.9 vs. 3.3%) and total bleeding (4.3 vs. 9.1%). However, this hypothesis could hardly be proved, since we couldn't get enough sample sizes. Additionally, we noted that there were six deaths occurring in Rothschild et al. (21) during hospitalization, which accounting for 43% (6/14) of all-cause deaths, and five of which were in the USAT group. In study of Rothschild et al. (21) longer duration and higher tPA dose were used in the USAT group compared to SCDT group, which may have contributed to the higher bleeding (13 vs. 8%) and mortality (8.1 vs. 2.8%). However, in our meta-analysis, USAT group had a trend of lower dosage and shorter infusion time of thrombolytic drugs than SCDT group. Therefore, a higher trend of bleeding complications and mortality was observed in the USAT group than in the SCDT group that may be not directly associated with dosage and infusion time of thrombolytic drugs in USAT in our study. The conclusions should be viewed with caution due to small sample size. Altogether, we need more real-world data to assess the causality between adverse clinical outcomes and thrombolytic drugs.

The SCDT group had a lower risk of bleeding than the USAT group, with no statistically significant difference in the incidence of total bleeding (4.3 vs. 9.1%) or major bleeding (1.9 vs. 3.3%). However, the SCDT group had significantly lower risk of bleeding than USAT group (9.4 vs. 2.3%, *P* = 0.04) in the subgroup of after-2019, while it was comparable between two catheters (8.5 vs. 7.8%, *P* = 0.93) before 2019. Comparing the trials before and after 2019, we found that, the USAT group received a lower dose over a shorter timeframe of tPA than SCDT in the trials before 2019, but no differences were found between the two catheters after 2019. USAT group had similar clinical outcomes with a lower dose and a shorter time of thrombolytic drugs without an increased risk of bleeding in the trials before 2019, since ultrasound assistance itself enhances the efficiency of thrombolytic therapy. Inappropriate dose and duration time of thrombolytic drugs in the USAT group attributed to an increased risk of bleeding. The OPTALYSE PE Trial had shown that a lower risk of bleeding, effective reduction in the pulmonary thrombotic load, improved right ventricular function was associated with a lower use of 4–8 mg tPA/2 h infusion with USAT in patients with stable hemodynamics of sPE. Perhaps a lower dosage and duration of thrombolytic drugs is appropriate for USAT (15). In fact, there is no consensus on the dosage and duration of thrombolytic drugs, and further clinical trials should attempt to select a lower dose of tPA in combination with USAT to minimize the risk of bleeding. Catheter size may be another potential association with bleeding. Catheter size was described in two trials in this study (17, 23). The EKOS USAT catheter was performed with a 5.2 F multilumen sideport infusion catheter with infusion lengths of 6–50 cm in (17), while 6–12 cm infusion length in (23). The length of infusion catheter is mainly depending on the length of the occlusion. The relationship between catheter size and bleeding was not explored in the two studies. In fact, no trials have been designed to deeply researched the relationship between catheter size and bleeding, which should be an innovation and focus for future studies.

Standard catheter-directed thrombolysis group was associated with a higher reduction of RV/LV than USAT group, and had significantly lower PASP in SCDT group at post-treatment. However, Miller score were similar between two groups. In SUNSET sPE trial, pulmonary obstruction score reduction was similar between USAT and SCDT, while RV/LV ratio reduction was significantly higher in SCDT than USAT (23). Therefore, the results of improved right ventricular function and lower PASP in the SCDT group than USAT group can't be explained by direct catheter action on the pulmonary arterial thrombus reduction. Regrettably, until now, there is no study to be designed for explaining this observation. Compared to sPE, patients with mPE may be more suitable for USAT. In Lin (17) with 25 cases of mPE, both the dose and duration time of tPA were significantly lower in the USAT group than those in the SCDT group and USAT group was associated with a lower risk of bleeding (0 vs. 21%). In our sensitivity analysis, when Lin (17) was excluded, we noted that SCDT had a lower bleeding than USAT (*P* = 0.032). Therefore, we may speculate that the addition of ultrasound truly adds an enhanced lytic effect in mPEs, while little in sPEs and with an increased risk of bleeding. More randomized trials are needed to be designed to verify above findings.

In our study, pulmonary obstruction score reduction was similar between two groups, while SCDT had shown an advantage at improving right ventricular function and lower LOS in hospital than USAT group. These results are consistent with SUNSET sPE Trial (23), which was designed to determine whether ultrasound-assisted thrombolysis (USAT) is superior to standard catheter-directed thrombolysis (SCDT) in pulmonary arterial thrombus reduction for patients with submassive pulmonary embolism (sPE). Additionally, we also found that USAT may enhanced lytic effect in mPE better than SCDT, while patients with sPE who are treated with USAT may be associated with an increased risk of bleeding than SCDT. Reduced dose and duration time of thrombolytic drugs with USAT may be next focus in future studies (14, 15, 23).

Longer LOS and 10 times costing of catheter in USAT group can increase costs for patients (26). Therefore, if USAT does not provide better efficacy and safety, it is not the first choice from an economic perspective.

## Limitations

Our study has several limitations. First, one of the seven articles included was an RCT trial and the other six were non-RCT trials, which provide underlying sources for heterogeneity in the meta-analysis; Second, the time span of the included studies is large; the longest time for patients to be included was 10 years, and the shortest was 3 years. With the improvement of adjuvant therapy techniques during this time and the continuous accumulation of surgeons' experience, complications and long-term follow-up results would likely be influenced. Third, the loading dose and the infusion rate of tPA were different between these studies, which may affect the incidence of clinical outcomes.

## Conclusion

In this study, both Ultrasound-assisted thrombolysis (USAT) and Standard catheter-directed thrombolysis (SCDT) have shown efficacy and safety for patients with acute pulmonary embolism (aPE). SCDT can improve right ventricular function better than USAT rapidly after treatment and had a lower length of stay (LOS) in hospital. However, due to small sample size, we cannot explore precise dose and time of tPA for patients with aPE at different risk levels. More larger sample size randomized control trials (RCT) will be needed to verify above conclusions.

## Author contributions

YL: obtained research funding, oversaw incubation and designed experiments. BS, JY, ZW, HZ, YC, and RC: collected data and supported work. All authors contributed to the article and approved the submitted version.

## Funding

This study was supported by Tang Du Yin Feng program (2021YFJH007).

## Conflict of interest

The authors declare that the research was conducted in the absence of any commercial or financial relationships that could be construed as a potential conflict of interest.

## Publisher's note

All claims expressed in this article are solely those of the authors and do not necessarily represent those of their affiliated organizations, or those of the publisher, the editors and the reviewers. Any product that may be evaluated in this article, or claim that may be made by its manufacturer, is not guaranteed or endorsed by the publisher.
